# Oxonium (dihydrogen 1-amino­ethane-1,1-diyldiphospho­nato-κ^2^ *N*,*O*)[hydrogen (1-amino-1-phosphono­ethyl)phospho­nato-κ^2^ *N*,*O*]palladium(II) trihydrate

**DOI:** 10.1107/S1600536810000930

**Published:** 2010-01-16

**Authors:** Anatolij Dudko, Vladimir Bon, Alexandra Kozachkova, Natalia Tsaryk, Vasily Pekhnyo

**Affiliations:** a Institute of General and Inorganic Chemistry, National Academy of Science Ukraine, Prospekt Palladina 32/34, Kyiv 03680, Ukraine

## Abstract

The title compound, (H_3_O)[Pd(C_2_H_7_NO_6_P_2_)(C_2_H_8_NO_6_P_2_)]·3H_2_O, was synthesized by the reaction of [Pd(H_2_O)_4_](NO_3_)_2_ with 1-amino­ethane-1,1-diyldiphospho­nic acid in aqueous solution. The asymmetric unit contains one mol­ecule of the complex existing as an anion, an oxonium counter-ion and three solvent water mol­ecules. The Pd^II^ ion occupies a position on a pseudo-twofold axis, which is not realized crystallographically. The slightly distorted square-planar coordination environment of the Pd^II^ ion consists of the O atoms from two phospho­nic acid groups and two N atoms of the amino groups in *cis* positions. The crystal structure displays N—H⋯O and O—H⋯O hydrogen bonding, which creates a wide three-dimensional network.

## Related literature

For general background and the medical use of organic diphospho­nic acids, see: Matczak-Jon & Videnova-Adrabinska (2005 ▶); Curic *et al.* (1996 ▶); Szabo *et al.* (2002 ▶). For related structures, see: Shkol’nikova *et al.* (1991 ▶).
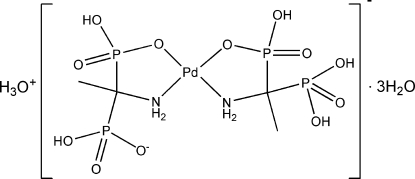


## Experimental

### 

#### Crystal data

(H_3_O)[Pd(C_2_H_7_NO_6_P_2_)(C_2_H_8_NO_6_P_2_)]·3H_2_O
*M* *_r_* = 586.53
Orthorhombic, 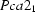

*a* = 9.9412 (2) Å
*b* = 9.0941 (2) Å
*c* = 19.9004 (3) Å
*V* = 1799.12 (6) Å^3^
*Z* = 4
Mo *K*α radiation
μ = 1.47 mm^−1^
*T* = 100 K
0.48 × 0.29 × 0.14 mm

#### Data collection

Bruker APEXII CCD diffractometer
Absorption correction: multi-scan (*SADABS*; Bruker, 2005 ▶) *T*_min_ = 0.536, *T*_max_ = 0.823
53680 measured reflections
5568 independent reflections
5545 reflections with *I* > 2σ(*I*)
*R*_int_ = 0.027

#### Refinement

*R*[*F* ^2^ > 2σ(*F* ^2^)] = 0.015
*wR*(*F* ^2^) = 0.041
*S* = 1.09
5568 reflections
301 parameters
6 restraints
H atoms treated by a mixture of independent and constrained refinement
Δρ_max_ = 0.80 e Å^−3^
Δρ_min_ = −0.61 e Å^−3^
Absolute structure: racemic twin (Flack, 1983 ▶), 2697 Friedel pairs
Flack parameter: 0.362 (11)

### 

Data collection: *APEX2* (Bruker, 2005 ▶); cell refinement: *SAINT* (Bruker, 2005 ▶); data reduction: *SAINT*; program(s) used to solve structure: *SHELXS97* (Sheldrick, 2008 ▶); program(s) used to refine structure: *SHELXL97* (Sheldrick, 2008 ▶); molecular graphics: *SHELXTL* (Sheldrick, 2008 ▶); software used to prepare material for publication: publCIF (Westrip, 2010 ▶).

## Supplementary Material

Crystal structure: contains datablocks I, global. DOI: 10.1107/S1600536810000930/hg2625sup1.cif

Structure factors: contains datablocks I. DOI: 10.1107/S1600536810000930/hg2625Isup2.hkl

Additional supplementary materials:  crystallographic information; 3D view; checkCIF report

## Figures and Tables

**Table 1:** Hydrogen-bond geometry (Å, °)

*D*—H⋯*A*	*D*—H	H⋯*A*	*D*⋯*A*	*D*—H⋯*A*
N1—H11N⋯O1^i^	0.83 (2)	2.28 (2)	3.0527 (16)	154 (2)
N1—H12N⋯O16	0.77 (2)	2.38 (2)	3.0647 (18)	149 (2)
N2—H21N⋯O7^i^	0.86 (2)	2.01 (2)	2.8245 (17)	159 (2)
N2—H22N⋯O10	0.80 (2)	2.53 (2)	2.9844 (17)	117.7 (19)
O2—H2O⋯O6^ii^	0.77 (2)	1.79 (2)	2.5582 (15)	174 (3)
O4—H4O⋯O11^iii^	0.73 (3)	1.94 (3)	2.6593 (16)	170 (3)
O13—H133⋯O5	1.06 (2)	1.40 (3)	2.4558 (16)	174 (2)
O8—H8O⋯O15	0.81 (2)	1.76 (2)	2.5607 (17)	173 (3)
O10—H10O⋯O16^iv^	0.83 (3)	1.80 (3)	2.6298 (17)	172 (3)
O12—H12O⋯O14	0.79 (2)	1.71 (2)	2.4608 (16)	159 (3)
O13—H131⋯O3^v^	0.80 (3)	1.79 (3)	2.5728 (16)	165 (2)
O13—H132⋯O9^vi^	0.89 (2)	1.62 (2)	2.5044 (16)	174 (2)
O14—H141⋯O3^vii^	0.79 (2)	1.96 (2)	2.7220 (16)	162 (2)
O14—H142⋯O11^viii^	0.80 (3)	1.90 (3)	2.6911 (17)	171 (3)
O15—H151⋯O4^ix^	0.82 (3)	2.18 (3)	2.9904 (18)	169 (3)
O15—H152⋯O6^iv^	0.79 (2)	1.97 (2)	2.7504 (17)	176 (3)
O16—H161⋯O14^ix^	0.76 (3)	2.19 (3)	2.8534 (16)	147 (3)
O16—H162⋯O8^x^	0.87 (3)	2.48 (3)	3.0109 (17)	120 (2)
O16—H162⋯O2	0.87 (3)	2.62 (3)	3.1809 (16)	123 (2)

Symmetry codes: (i) 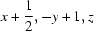; (ii) 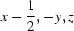; (iii) 
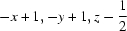
; (iv) 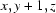; (v) 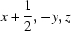; (vi) 
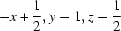
; (vii) 
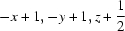
; (viii) 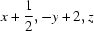; (ix) 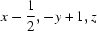; (x) 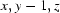.

**Fig. 1.**
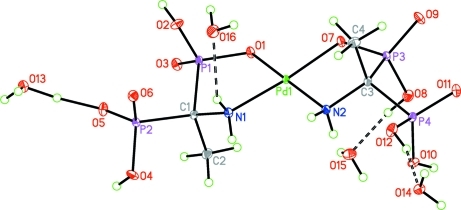
The title compound showing 50% probability displacement ellipsoids for the non-H atoms. Dashed lines indicate hydrogen bonds.

**Fig. 2.**
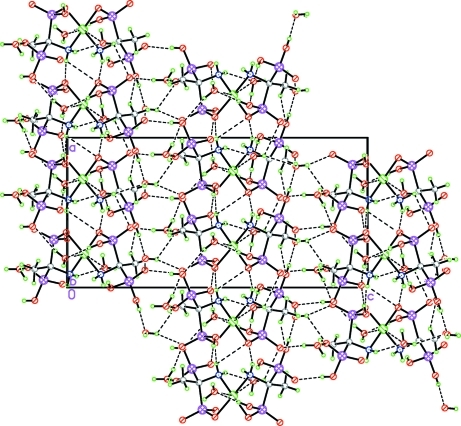
Crystal packing of title compound, projection down the b axis. Dashed lines indicate hydrogen bonds.

## supplementary crystallographic information

### Comment

During the last decade, there has been a growing interest in the study of organic diphosphonic acids owing to their potentially very powerful chelating properties used in metal extractions and are tested by the pharmaceutical industry for use as efficient drugs preventing calcification and inhibiting bone resorption (Matczak-Jon & Videnova-Adrabinska, 2005). Diphosphonic acids and their metal complexes are used in the treatment of Pagets disease, osteoporosis and tumoral osteolysis (Szabo *et al.*, 2002). Also in the last years, there has been a surge of interest in palladium complexes as a perspective antitumor preparation (Curic *et al.*, 1996).

The title compound crystallized in non-centrosymmetric space group *Pca2_1_* with Flack parameter equal 0.362 (11), which indicate the presence of racemic twin in the structure (Flack, 1983). The asymmetric unit of title compound contains one formula unit, which exists as a complex anion and oxonium cation, which are bonding together *via* strong H-bond (Fig.1, Table 1). The Pd atom occupies a position on the pseudo twofold axis and shows slightly distorted square-planar coordination environment, which consists of O atoms from two phosphonic groups and two N atoms of amino group, located in *cis* position. The crystal structure displays N—H···O and O—H···O hydrogen bonding, which creates a three-dimensional network (Table 1, Fig.2). Hydrogen bonds often play a dominant role in crystal engineering (Matczak-Jon & Videnova-Adrabinska, 2005) because they combine the desirable attributes of specificity, strength and directionality.

### Experimental

Aqueous solution of AgNO_3_ (0,4076 g, 0,24 mmol) was added to the solution of PdCl_2_ (0,063 g, 0,6 mmol) in 12 ml of hydrochloric acid. The solution was stirred at 3–4 °C and protected from light for 30 min. The AgCl precipitated and was filtered off. The dark red solution turned yellow and than 1-aminoethane-1,1-diyldiphosphonic acid (0,2459 g, 0,12 mmol) was added in one portion. The solution was stirred for 1 h at 3–4 °C and left staying overnight at room temperature. The solvent was removed under reduced pressure leaving a pale yellow solid, which was washed twice with methanol and diethyl ether and dried under vacuum. Suitable single crystals of title compound were produced by slow evaporation of a water solution at room temperature. A pale yellow rectangular crystal was used for data collection.

### Refinement

H atoms bonded to N and O atoms were located in a difference map and refined with constrained *U*_iso_(H) = 1.2*U*_eq_(N) and *U*_iso_(H) = 1.5*U*_eq_(O). Other H atoms were positioned geometrically and refined using riding model with C—H = 0.98 Å for CH_3_ [*U*_iso_(H) = 1.5*U*_eq_(C)]. Several restraints were used in the final refinement for improving the O—H distances O2—H2O, O8—H8O, O12—H12O, O14—H1 41, O15—H152 onto reasonable values.

### Crystal data

**Table:**

(H_3_O)[Pd(C_2_H_7_NO_6_P_2_)(C_2_H_8_NO_6_P_2_)]·3H_2_O	*D*_x_ = 2.165 Mg m^−^^3^
*M**_r_* = 586.53	Melting point: 542 K
Orthorhombic, *P**c**a*2_1_	Mo *K*α radiation, λ = 0.71073 Å
Hall symbol: P 2c -2ac	Cell parameters from 9199 reflections
*a* = 9.9412 (2) Å	θ = 3.0–30.7°
*b* = 9.0941 (2) Å	µ = 1.47 mm^−^^1^
*c* = 19.9004 (3) Å	*T* = 100 K
*V* = 1799.12 (6) Å^3^	Prism, yellow
*Z* = 4	0.48 × 0.29 × 0.14 mm
*F*(000) = 1184	

### Data collection

**Table:**

Bruker APEXII CCD diffractometer	5568 independent reflections
Radiation source: fine-focus sealed tube	5545 reflections with *I* > 2σ(*I*)
graphite	*R*_int_ = 0.027
Detector resolution: 8.26 pixels mm^-1^	θ_max_ = 30.7°, θ_min_ = 2.1°
φ and ω scans	*h* = −14→14
Absorption correction: multi-scan (*SADABS*; Bruker, 2005)	*k* = −13→13
*T*_min_ = 0.536, *T*_max_ = 0.823	*l* = −28→28
53680 measured reflections	

### Refinement

**Table:**

Refinement on *F*^2^	Secondary atom site location: difference Fourier map
Least-squares matrix: full	Hydrogen site location: inferred from neighbouring sites
*R*[*F*^2^ > 2σ(*F*^2^)] = 0.015	H atoms treated by a mixture of independent and constrained refinement
*wR*(*F*^2^) = 0.041	*w* = 1/[σ^2^(*F*_o_^2^) + (0.0254*P*)^2^ + 0.6821*P*] where *P* = (*F*_o_^2^ + 2*F*_c_^2^)/3
*S* = 1.09	(Δ/σ)_max_ = 0.001
5568 reflections	Δρ_max_ = 0.80 e Å^−^^3^
301 parameters	Δρ_min_ = −0.61 e Å^−^^3^
6 restraints	Absolute structure: racemic twin (Flack, 1983), with how many Friedel pairs?
Primary atom site location: structure-invariant direct methods	Flack parameter: 0.362 (11)

### Special details

**Table:**

Geometry. All e.s.d.'s (except the e.s.d. in the dihedral angle between two l.s. planes) are estimated using the full covariance matrix. The cell e.s.d.'s are taken into account individually in the estimation of e.s.d.'s in distances, angles and torsion angles; correlations between e.s.d.'s in cell parameters are only used when they are defined by crystal symmetry. An approximate (isotropic) treatment of cell e.s.d.'s is used for estimating e.s.d.'s involving l.s. planes.
Refinement. Refinement of *F*^2^ against ALL reflections. The weighted *R*-factor *wR* and goodness of fit *S* are based on *F*^2^, conventional *R*-factors *R* are based on *F*, with *F* set to zero for negative *F*^2^. The threshold expression of *F*^2^ > σ(*F*^2^) is used only for calculating *R*-factors(gt) *etc*. and is not relevant to the choice of reflections for refinement. *R*-factors based on *F*^2^ are statistically about twice as large as those based on *F*, and *R*- factors based on ALL data will be even larger.

### Fractional atomic coordinates and isotropic or equivalent isotropic displacement parameters (Å^2^)

**Table:**

	*x*	*y*	*z*	*U*_iso_*/*U*_eq_	
Pd1	0.277544 (8)	0.490311 (10)	0.552262 (8)	0.00533 (3)	
P1	0.18198 (3)	0.27187 (4)	0.450244 (18)	0.00635 (6)	
P2	0.45406 (3)	0.13836 (4)	0.414989 (18)	0.00657 (6)	
P3	0.18727 (3)	0.72538 (4)	0.648914 (17)	0.00648 (6)	
P4	0.46193 (3)	0.80633 (4)	0.704329 (19)	0.00694 (6)	
C1	0.36318 (13)	0.30710 (15)	0.43864 (7)	0.0064 (2)	
C2	0.37971 (15)	0.42927 (16)	0.38612 (7)	0.0101 (2)	
H2A	0.3247	0.5142	0.3990	0.015*	
H2B	0.3506	0.3930	0.3421	0.015*	
H2C	0.4744	0.4587	0.3838	0.015*	
C3	0.35636 (14)	0.65907 (15)	0.67191 (7)	0.0068 (2)	
C4	0.34278 (15)	0.53574 (17)	0.72415 (7)	0.0107 (2)	
H4A	0.2757	0.4642	0.7088	0.016*	
H4B	0.3142	0.5777	0.7672	0.016*	
H4C	0.4298	0.4866	0.7298	0.016*	
N1	0.41461 (12)	0.36316 (14)	0.50470 (6)	0.0071 (2)	
H11N	0.488 (2)	0.403 (3)	0.4966 (12)	0.009*	
H12N	0.429 (2)	0.295 (3)	0.5263 (11)	0.009*	
N2	0.41624 (12)	0.59683 (14)	0.60867 (6)	0.0071 (2)	
H21N	0.487 (2)	0.545 (3)	0.6173 (12)	0.009*	
H22N	0.447 (2)	0.661 (3)	0.5861 (11)	0.009*	
O1	0.13407 (10)	0.40368 (12)	0.49205 (5)	0.00857 (18)	
O2	0.17592 (11)	0.13034 (12)	0.49408 (6)	0.0109 (2)	
H2O	0.121 (2)	0.072 (2)	0.4877 (12)	0.016*	
O3	0.11271 (12)	0.25861 (13)	0.38397 (6)	0.0100 (2)	
O4	0.59246 (11)	0.19312 (13)	0.38559 (6)	0.0108 (2)	
H4O	0.595 (2)	0.180 (3)	0.3495 (13)	0.016*	
O5	0.37046 (11)	0.06419 (12)	0.36199 (5)	0.01071 (19)	
H133	0.379 (2)	−0.084 (3)	0.3421 (12)	0.016*	
O6	0.48312 (10)	0.05260 (13)	0.47795 (6)	0.00955 (19)	
O7	0.13394 (10)	0.60237 (12)	0.60317 (5)	0.00854 (19)	
O8	0.20867 (11)	0.86805 (13)	0.60679 (6)	0.0109 (2)	
H8O	0.238 (2)	0.846 (3)	0.5705 (9)	0.016*	
O9	0.10146 (11)	0.75447 (13)	0.70822 (6)	0.0119 (2)	
O10	0.50545 (12)	0.90097 (12)	0.64312 (6)	0.01182 (19)	
H10O	0.470 (3)	0.982 (3)	0.6345 (16)	0.018*	
O11	0.38539 (11)	0.88643 (13)	0.75747 (5)	0.01041 (19)	
O12	0.58885 (11)	0.72475 (14)	0.72731 (6)	0.0118 (2)	
H12O	0.649 (2)	0.778 (3)	0.7377 (12)	0.018*	
O13	0.38258 (11)	−0.19735 (12)	0.33187 (5)	0.00984 (19)	
H131	0.447 (3)	−0.227 (3)	0.3520 (13)	0.015*	
H132	0.382 (2)	−0.212 (3)	0.2878 (12)	0.015*	
O14	0.80746 (11)	0.83022 (13)	0.75959 (5)	0.01002 (18)	
H141	0.813 (2)	0.801 (2)	0.7968 (9)	0.015*	
H142	0.822 (3)	0.917 (3)	0.7586 (12)	0.015*	
O15	0.30815 (12)	0.81984 (13)	0.49016 (6)	0.0136 (2)	
H151	0.257 (3)	0.820 (3)	0.4577 (13)	0.020*	
H152	0.356 (2)	0.888 (2)	0.4850 (13)	0.020*	
O16	0.37176 (13)	0.14546 (13)	0.62029 (6)	0.0138 (2)	
H161	0.376 (3)	0.179 (3)	0.6551 (13)	0.021*	
H162	0.287 (3)	0.126 (3)	0.6135 (13)	0.021*	

### Atomic displacement parameters (Å^2^)

**Table:**

	*U*^11^	*U*^22^	*U*^33^	*U*^12^	*U*^13^	*U*^23^
Pd1	0.00476 (4)	0.00531 (4)	0.00592 (4)	0.00017 (3)	−0.00038 (4)	−0.00138 (4)
P1	0.00546 (15)	0.00627 (15)	0.00734 (15)	0.00002 (11)	−0.00077 (11)	−0.00155 (12)
P2	0.00633 (13)	0.00681 (15)	0.00657 (14)	0.00094 (11)	−0.00007 (11)	−0.00088 (12)
P3	0.00557 (14)	0.00721 (15)	0.00667 (15)	0.00051 (11)	0.00001 (11)	−0.00196 (12)
P4	0.00715 (14)	0.00637 (15)	0.00732 (14)	−0.00025 (11)	−0.00118 (11)	−0.00102 (12)
C1	0.0067 (5)	0.0059 (5)	0.0066 (5)	−0.0002 (4)	−0.0007 (4)	−0.0005 (4)
C2	0.0112 (6)	0.0077 (6)	0.0114 (6)	0.0000 (5)	0.0000 (5)	0.0017 (5)
C3	0.0083 (5)	0.0057 (5)	0.0064 (5)	0.0000 (4)	−0.0004 (4)	−0.0008 (4)
C4	0.0113 (6)	0.0111 (6)	0.0098 (6)	−0.0008 (5)	−0.0014 (5)	0.0030 (5)
N1	0.0056 (5)	0.0080 (5)	0.0078 (5)	0.0005 (4)	−0.0012 (4)	−0.0016 (4)
N2	0.0063 (5)	0.0073 (5)	0.0077 (5)	−0.0007 (4)	0.0001 (4)	−0.0017 (4)
O1	0.0051 (4)	0.0104 (5)	0.0102 (4)	0.0008 (3)	−0.0014 (3)	−0.0049 (4)
O2	0.0090 (4)	0.0095 (5)	0.0141 (5)	−0.0031 (4)	−0.0020 (4)	0.0026 (4)
O3	0.0075 (4)	0.0135 (5)	0.0092 (5)	0.0005 (4)	−0.0027 (3)	−0.0034 (4)
O4	0.0082 (4)	0.0152 (5)	0.0089 (5)	−0.0004 (4)	0.0031 (3)	−0.0006 (4)
O5	0.0125 (5)	0.0090 (5)	0.0106 (5)	0.0019 (4)	−0.0032 (4)	−0.0029 (4)
O6	0.0101 (5)	0.0085 (5)	0.0101 (5)	0.0021 (4)	−0.0002 (3)	0.0009 (4)
O7	0.0063 (4)	0.0100 (5)	0.0093 (5)	0.0008 (3)	−0.0003 (3)	−0.0042 (4)
O8	0.0127 (5)	0.0082 (5)	0.0119 (5)	0.0009 (3)	−0.0006 (4)	0.0011 (4)
O9	0.0088 (4)	0.0175 (5)	0.0094 (5)	0.0016 (4)	0.0021 (4)	−0.0047 (4)
O10	0.0136 (5)	0.0086 (5)	0.0133 (5)	−0.0024 (4)	0.0032 (4)	0.0017 (4)
O11	0.0127 (5)	0.0089 (5)	0.0096 (4)	0.0021 (4)	−0.0006 (4)	−0.0024 (4)
O12	0.0089 (5)	0.0097 (5)	0.0167 (5)	0.0004 (4)	−0.0051 (4)	−0.0018 (4)
O13	0.0093 (4)	0.0108 (5)	0.0094 (5)	0.0009 (4)	−0.0020 (4)	−0.0014 (4)
O14	0.0100 (4)	0.0101 (5)	0.0099 (5)	−0.0014 (4)	−0.0005 (4)	0.0007 (4)
O15	0.0148 (5)	0.0129 (5)	0.0131 (5)	−0.0017 (4)	0.0014 (4)	0.0033 (4)
O16	0.0180 (5)	0.0133 (5)	0.0101 (5)	−0.0017 (4)	−0.0008 (4)	−0.0002 (4)

### Geometric parameters (Å, °)

**Table:**

Pd1—N1	2.0223 (12)	C3—N2	1.5028 (18)
Pd1—O1	2.0226 (10)	C3—C4	1.535 (2)
Pd1—N2	2.0247 (12)	C4—H4A	0.9800
Pd1—O7	2.0256 (10)	C4—H4B	0.9800
P1—O3	1.4926 (12)	C4—H4C	0.9800
P1—O1	1.5349 (11)	N1—H11N	0.83 (2)
P1—O2	1.5560 (12)	N1—H12N	0.77 (2)
P1—C1	1.8442 (14)	N2—H21N	0.86 (2)
P2—O5	1.5027 (11)	N2—H22N	0.80 (2)
P2—O6	1.5039 (12)	O2—H2O	0.774 (16)
P2—O4	1.5759 (11)	O4—H4O	0.73 (3)
P2—C1	1.8418 (14)	O5—H133	1.40 (3)
P3—O9	1.4801 (12)	O8—H8O	0.805 (16)
P3—O7	1.5366 (11)	O10—H10O	0.83 (3)
P3—O8	1.5593 (12)	O12—H12O	0.791 (17)
P3—C3	1.8435 (14)	O13—H133	1.06 (2)
P4—O11	1.4926 (11)	O13—H131	0.80 (3)
P4—O12	1.5335 (12)	O13—H132	0.89 (2)
P4—O10	1.5530 (12)	O14—H141	0.788 (16)
P4—C3	1.8196 (14)	O14—H142	0.80 (3)
C1—N1	1.4998 (18)	O15—H151	0.82 (3)
C1—C2	1.5342 (19)	O15—H152	0.785 (16)
C2—H2A	0.9800	O16—H161	0.76 (3)
C2—H2B	0.9800	O16—H162	0.87 (3)
C2—H2C	0.9800		
			
N1—Pd1—O1	88.58 (5)	H2A—C2—H2C	109.5
N1—Pd1—N2	94.26 (5)	H2B—C2—H2C	109.5
O1—Pd1—N2	174.28 (5)	N2—C3—C4	109.07 (11)
N1—Pd1—O7	175.31 (5)	N2—C3—P4	110.22 (9)
O1—Pd1—O7	89.73 (4)	C4—C3—P4	110.38 (9)
N2—Pd1—O7	87.82 (5)	N2—C3—P3	106.05 (9)
O3—P1—O1	113.50 (6)	C4—C3—P3	109.08 (10)
O3—P1—O2	114.25 (7)	P4—C3—P3	111.91 (7)
O1—P1—O2	109.28 (6)	C3—C4—H4A	109.5
O3—P1—C1	110.74 (6)	C3—C4—H4B	109.5
O1—P1—C1	103.61 (6)	H4A—C4—H4B	109.5
O2—P1—C1	104.58 (6)	C3—C4—H4C	109.5
O5—P2—O6	117.27 (7)	H4A—C4—H4C	109.5
O5—P2—O4	111.35 (7)	H4B—C4—H4C	109.5
O6—P2—O4	107.79 (6)	C1—N1—Pd1	112.02 (8)
O5—P2—C1	106.38 (6)	C1—N1—H11N	106.1 (16)
O6—P2—C1	108.26 (6)	Pd1—N1—H11N	115.9 (16)
O4—P2—C1	105.06 (6)	C1—N1—H12N	106.4 (17)
O9—P3—O7	113.81 (6)	Pd1—N1—H12N	108.9 (17)
O9—P3—O8	111.01 (7)	H11N—N1—H12N	107 (2)
O7—P3—O8	109.53 (6)	C3—N2—Pd1	112.01 (8)
O9—P3—C3	112.71 (6)	C3—N2—H21N	111.1 (16)
O7—P3—C3	102.92 (6)	Pd1—N2—H21N	113.9 (16)
O8—P3—C3	106.34 (6)	C3—N2—H22N	110.5 (16)
O11—P4—O12	116.37 (6)	Pd1—N2—H22N	107.3 (16)
O11—P4—O10	115.30 (7)	H21N—N2—H22N	101 (2)
O12—P4—O10	105.83 (7)	P1—O1—Pd1	113.98 (6)
O11—P4—C3	108.45 (7)	P1—O2—H2O	120.4 (18)
O12—P4—C3	102.96 (7)	P2—O4—H4O	110.0 (19)
O10—P4—C3	106.88 (6)	P2—O5—H133	126.4 (10)
N1—C1—C2	108.33 (11)	P3—O7—Pd1	114.80 (6)
N1—C1—P2	109.88 (9)	P3—O8—H8O	108.8 (17)
C2—C1—P2	112.13 (9)	P4—O10—H10O	122 (2)
N1—C1—P1	106.40 (9)	P4—O12—H12O	113.7 (19)
C2—C1—P1	108.40 (9)	H133—O13—H131	105 (2)
P2—C1—P1	111.49 (7)	H133—O13—H132	110 (2)
C1—C2—H2A	109.5	H131—O13—H132	116 (2)
C1—C2—H2B	109.5	H141—O14—H142	110 (2)
H2A—C2—H2B	109.5	H151—O15—H152	106 (3)
C1—C2—H2C	109.5	H161—O16—H162	106 (3)
			
O5—P2—C1—N1	162.48 (9)	O7—P3—C3—N2	42.06 (10)
O6—P2—C1—N1	35.62 (11)	O8—P3—C3—N2	−73.07 (10)
O4—P2—C1—N1	−79.34 (10)	O9—P3—C3—C4	47.74 (11)
O5—P2—C1—C2	−76.99 (11)	O7—P3—C3—C4	−75.28 (10)
O6—P2—C1—C2	156.15 (10)	O8—P3—C3—C4	169.59 (9)
O4—P2—C1—C2	41.19 (11)	O9—P3—C3—P4	−74.69 (9)
O5—P2—C1—P1	44.78 (9)	O7—P3—C3—P4	162.28 (7)
O6—P2—C1—P1	−82.09 (8)	O8—P3—C3—P4	47.16 (9)
O4—P2—C1—P1	162.95 (7)	C2—C1—N1—Pd1	80.78 (11)
O3—P1—C1—N1	163.27 (9)	P2—C1—N1—Pd1	−156.42 (6)
O1—P1—C1—N1	41.23 (10)	P1—C1—N1—Pd1	−35.59 (11)
O2—P1—C1—N1	−73.22 (10)	O1—Pd1—N1—C1	17.73 (10)
O3—P1—C1—C2	46.95 (11)	N2—Pd1—N1—C1	−157.30 (10)
O1—P1—C1—C2	−75.08 (10)	C4—C3—N2—Pd1	79.72 (12)
O2—P1—C1—C2	170.47 (9)	P4—C3—N2—Pd1	−158.94 (6)
O3—P1—C1—P2	−76.95 (9)	P3—C3—N2—Pd1	−37.63 (10)
O1—P1—C1—P2	161.02 (7)	N1—Pd1—N2—C3	−156.26 (9)
O2—P1—C1—P2	46.57 (8)	O7—Pd1—N2—C3	19.52 (9)
O11—P4—C3—N2	167.68 (9)	O3—P1—O1—Pd1	−148.99 (7)
O12—P4—C3—N2	−68.45 (10)	O2—P1—O1—Pd1	82.21 (7)
O10—P4—C3—N2	42.79 (11)	C1—P1—O1—Pd1	−28.83 (8)
O11—P4—C3—C4	−71.76 (11)	N1—Pd1—O1—P1	10.10 (7)
O12—P4—C3—C4	52.11 (11)	O7—Pd1—O1—P1	−165.52 (7)
O10—P4—C3—C4	163.35 (10)	O9—P3—O7—Pd1	−150.89 (7)
O11—P4—C3—P3	49.93 (9)	O8—P3—O7—Pd1	84.19 (7)
O12—P4—C3—P3	173.79 (7)	C3—P3—O7—Pd1	−28.61 (8)
O10—P4—C3—P3	−74.96 (9)	O1—Pd1—O7—P3	−165.75 (7)
O9—P3—C3—N2	165.08 (9)	N2—Pd1—O7—P3	9.09 (7)

### Hydrogen-bond geometry (Å, °)

**Table:**

*D*—H···*A*	*D*—H	H···*A*	*D*···*A*	*D*—H···*A*
N1—H11N···O1^i^	0.83 (2)	2.28 (2)	3.0527 (16)	154 (2)
N1—H12N···O16	0.77 (2)	2.38 (2)	3.0647 (18)	149 (2)
N2—H21N···O7^i^	0.86 (2)	2.01 (2)	2.8245 (17)	159 (2)
N2—H22N···O10	0.80 (2)	2.53 (2)	2.9844 (17)	117.7 (19)
O2—H2O···O6^ii^	0.77 (2)	1.79 (2)	2.5582 (15)	174 (3)
O4—H4O···O11^iii^	0.73 (3)	1.94 (3)	2.6593 (16)	170 (3)
O13—H133···O5	1.06 (2)	1.40 (3)	2.4558 (16)	174 (2)
O8—H8O···O15	0.81 (2)	1.76 (2)	2.5607 (17)	173 (3)
O10—H10O···O16^iv^	0.83 (3)	1.80 (3)	2.6298 (17)	172 (3)
O12—H12O···O14	0.79 (2)	1.71 (2)	2.4608 (16)	159 (3)
O13—H131···O3^v^	0.80 (3)	1.79 (3)	2.5728 (16)	165 (2)
O13—H132···O9^vi^	0.89 (2)	1.62 (2)	2.5044 (16)	174 (2)
O14—H141···O3^vii^	0.79 (2)	1.96 (2)	2.7220 (16)	162 (2)
O14—H142···O11^viii^	0.80 (3)	1.90 (3)	2.6911 (17)	171 (3)
O15—H151···O4^ix^	0.82 (3)	2.18 (3)	2.9904 (18)	169 (3)
O15—H152···O6^iv^	0.79 (2)	1.97 (2)	2.7504 (17)	176 (3)
O16—H161···O14^ix^	0.76 (3)	2.19 (3)	2.8534 (16)	147 (3)
O16—H162···O8^x^	0.87 (3)	2.48 (3)	3.0109 (17)	120 (2)
O16—H162···O2	0.87 (3)	2.62 (3)	3.1809 (16)	123 (2)

Symmetry codes: (i) *x*+1/2, −*y*+1, *z*; (ii) *x*−1/2, −*y*, *z*; (iii) −*x*+1, −*y*+1, *z*−1/2; (iv) *x*, *y*+1, *z*; (v) *x*+1/2, −*y*, *z*; (vi) −*x*+1/2, *y*−1, *z*−1/2; (vii) −*x*+1, −*y*+1, *z*+1/2; (viii) *x*+1/2, −*y*+2, *z*; (ix) *x*−1/2, −*y*+1, *z*; (x) *x*, *y*−1, *z*.
